# Supplementary material for: Altered microRNA Transcriptome in Cultured Human Liver Cells upon Infection with Ebola Virus
Source: Int J Mol Sci. 2021 Apr 6;22(7):3792. doi: 10.3390/ijms22073792 (PMC8038836; doi:10.3390/ijms22073792)
Supplement: Supplementary file 1 [file ijms-22-03792-s001.zip › Supplementary_File/C_ GO_Analysis_Results/16-30nt_go_Reston-24h-Huh7_vs_Control-24h-Huh7_up.mature_mirna_targets/MF_result(Human).html]

| GO.ID | Term | Ontology | Count | Pop.Hits | List.Total | Pop.Total | Fold.Enrichment | Pvalue | FDR | Enrichment.Score | Gene.Ratio | GENES |
| --- | --- | --- | --- | --- | --- | --- | --- | --- | --- | --- | --- | --- |
| GO:0000981 | RNA polymerase II transcription factor activity, sequence-specific DNA binding | Molecular function | 72 | 1108 | 582 | 17548 | 1.95928393315717 | 2.97884019413107e-08 | 3.86057689159387e-05 | 7.52595279476033 | 0.123711340206186 | FOSL1//CEBPB//ELK3//ESR1//TFEC//PTF1A//GATA2//IRF1//AR//MEOX2//MYB//MYBL1//MYOG//NEUROD1//NFIA//NHLH2//NR4A2//OTX2//POU3F2//POU4F1//RFX4//SOX4//CARF//KLF7//TSHZ1//CC2D1B//EN1//MYT1L//RCOR1//ZFPM2//ZNF217//BHLHE41//BHLHE40//ARX//PURG//JARID2//ZBTB4//ARID5B//FOXJ3//FOXF2//MACC1//IRF2//MAF//VGLL1//RFX3//PPARG//ZBTB18//ZNF268//KLF12//EMX2//HBP1//HIVEP2//HOXA3//HOXB6//HOXC8//HOXD1//BOLA3//NR3C2//NEUROG1//NFE2//TRERF1//MIER1//DMRTA1//RFX7//ZNF3//ZNF131//TSC22D4//LCOR//RHOXF2//RUNX3//CBFB//ST18// |
| GO:0001067 | regulatory region nucleic acid binding | Molecular function | 59 | 878 | 582 | 17548 | 2.02610587949808 | 1.92616971839534e-07 | 0.000124815797752018 | 6.71530544903121 | 0.101374570446735 | BAHD1//ZNF568//NFE2//PPARG//SOX4//CEBPB//ZMYND11//FOXF2//GATA2//PURG//MACC1//IRF2//JARID2//MYOG//NHLH2//NR4A2//KLF3//ZBTB4//ARID5B//RUNX3//CC2D1B//ELK3//EN1//ESR1//TFEC//IRF1//AR//MEOX2//MYB//MYBL1//NFIA//OTX2//POU3F2//RFX3//RFX4//RFX7//SMARCD2//ZNF217//BHLHE41//CARF//FOSL1//KLF7//GADD45A//ARX//PTF1A//POU4F1//ST18//MBNL1//SLC2A4RG//KLF17//RCOR1//HIVEP2//ARID4B//TRERF1//TNF//TBL1XR1//NEUROD1//NEUROG1//BHLHE40// |
| GO:0044212 | transcription regulatory region DNA binding | Molecular function | 58 | 877 | 582 | 17548 | 1.99403621374022 | 4.17676649371376e-07 | 0.000180436312528434 | 6.3791598037213 | 0.0996563573883162 | BAHD1//ZNF568//NFE2//PPARG//SOX4//CEBPB//ZMYND11//FOXF2//GATA2//PURG//MACC1//IRF2//JARID2//MYOG//NHLH2//NR4A2//KLF3//ZBTB4//ARID5B//RUNX3//CC2D1B//ELK3//EN1//ESR1//TFEC//IRF1//AR//MEOX2//MYB//MYBL1//NFIA//OTX2//POU3F2//RFX3//RFX4//RFX7//SMARCD2//ZNF217//BHLHE41//CARF//FOSL1//KLF7//GADD45A//ARX//PTF1A//POU4F1//ST18//SLC2A4RG//NEUROD1//NEUROG1//BHLHE40//KLF17//RCOR1//HIVEP2//ARID4B//TRERF1//TNF//TBL1XR1// |
| GO:0003700 | DNA binding transcription factor activity | Molecular function | 86 | 1551 | 582 | 17548 | 1.67182684489111 | 1.35503272957145e-06 | 0.000385640586928284 | 5.86805021467843 | 0.147766323024055 | TSHZ1//ZBTB18//CEBPB//ZNF268//KLF12//ARX//CC2D1B//ELK3//EMX2//EN1//ESR1//TFEC//FOXJ3//FOXF2//MYT1L//RCOR1//ZFPM2//PTF1A//GATA2//HBP1//HIVEP2//HOXA3//HOXB6//HOXC8//HOXD1//IRF1//IRF2//AR//BOLA3//MAF//MEOX2//NR3C2//MYB//MYBL1//MYOG//NEUROD1//NEUROG1//NFIA//NFE2//NHLH2//NR4A2//OTX2//POU3F2//POU4F1//PPARG//TRERF1//ZBTB4//MIER1//RFX3//RFX4//DMRTA1//RFX7//SOX4//ZNF3//ZNF131//BHLHE41//FOSL1//TSC22D4//LCOR//RHOXF2//BHLHE40//RUNX3//CBFB//ST18//CARF//KLF7//ZNF217//PURG//JARID2//ARID5B//MACC1//VGLL1//KLF17//ZNF782//ZFP30//CNOT7//ZNF568//KLF3//SLC2A4RG//ZNF630//ZNF134//ZNF227//ZNF655//BLZF1//CBL//BTAF1// |
| GO:0000976 | transcription regulatory region sequence-specific DNA binding | Molecular function | 50 | 740 | 582 | 17548 | 2.03724342899601 | 1.48781090635912e-06 | 0.000385640586928284 | 5.82745226203157 | 0.0859106529209622 | CEBPB//ZMYND11//FOXF2//GATA2//PURG//MACC1//IRF2//JARID2//MYOG//NHLH2//NR4A2//KLF3//PPARG//ZBTB4//ARID5B//RUNX3//CC2D1B//ELK3//EN1//ESR1//TFEC//IRF1//AR//MEOX2//MYB//MYBL1//NFIA//OTX2//POU3F2//RFX3//RFX4//RFX7//SMARCD2//ZNF217//BHLHE41//CARF//FOSL1//KLF7//GADD45A//ARX//PTF1A//POU4F1//NFE2//SOX4//SLC2A4RG//NEUROD1//NEUROG1//BHLHE40//BAHD1//ZNF568// |
| GO:0000982 | transcription factor activity, RNA polymerase II proximal promoter sequence-specific DNA binding | Molecular function | 33 | 415 | 582 | 17548 | 2.3975655198112 | 3.45638949501646e-06 | 0.000721189112967356 | 5.46137732348334 | 0.0567010309278351 | CEBPB//ELK3//ESR1//TFEC//PTF1A//GATA2//IRF1//AR//MEOX2//MYB//MYBL1//MYOG//NEUROD1//NFIA//NHLH2//NR4A2//OTX2//POU3F2//POU4F1//RFX4//SOX4//CARF//FOSL1//KLF7//TSHZ1//CC2D1B//EN1//MYT1L//RCOR1//ZFPM2//ZNF217//BHLHE41//BHLHE40// |
| GO:0000977 | RNA polymerase II regulatory region sequence-specific DNA binding | Molecular function | 45 | 662 | 582 | 17548 | 2.04955305696577 | 4.33651911993045e-06 | 0.000721189112967356 | 5.36285873444327 | 0.077319587628866 | CEBPB//CC2D1B//ELK3//EN1//ESR1//TFEC//GATA2//IRF1//AR//MEOX2//MYB//MYBL1//MYOG//NFIA//NHLH2//NR4A2//OTX2//POU3F2//RFX3//RFX4//RFX7//SMARCD2//ZNF217//BHLHE41//CARF//FOSL1//KLF7//GADD45A//ARX//PTF1A//POU4F1//NEUROD1//NEUROG1//BHLHE40//ZMYND11//FOXF2//PURG//MACC1//IRF2//JARID2//KLF3//PPARG//ZBTB4//ARID5B//RUNX3// |
| GO:0001012 | RNA polymerase II regulatory region DNA binding | Molecular function | 45 | 665 | 582 | 17548 | 2.04030695294938 | 4.87631674171582e-06 | 0.000721189112967356 | 5.31190809248932 | 0.077319587628866 | CEBPB//ZMYND11//FOXF2//GATA2//PURG//MACC1//IRF2//JARID2//MYOG//NHLH2//NR4A2//KLF3//PPARG//ZBTB4//ARID5B//RUNX3//CC2D1B//ELK3//EN1//ESR1//TFEC//IRF1//AR//MEOX2//MYB//MYBL1//NFIA//OTX2//POU3F2//RFX3//RFX4//RFX7//SMARCD2//ZNF217//BHLHE41//CARF//FOSL1//KLF7//GADD45A//ARX//PTF1A//POU4F1//NEUROD1//NEUROG1//BHLHE40// |
| GO:0140110 | transcription regulator activity | Molecular function | 99 | 1921 | 582 | 17548 | 1.55386208858144 | 5.00825772893997e-06 | 0.000721189112967356 | 5.30031333008189 | 0.170103092783505 | TSHZ1//ZBTB18//CEBPB//ZNF268//KLF12//ARX//CC2D1B//ELK3//EMX2//EN1//ESR1//TFEC//FOXJ3//FOXF2//MYT1L//RCOR1//ZFPM2//PTF1A//GATA2//HBP1//HIVEP2//HOXA3//HOXB6//HOXC8//HOXD1//IRF1//IRF2//AR//BOLA3//MAF//MEOX2//NR3C2//MYB//MYBL1//MYOG//NEUROD1//NEUROG1//NFIA//NFE2//NHLH2//NR4A2//OTX2//POU3F2//POU4F1//PPARG//TRERF1//ZBTB4//MIER1//RFX3//RFX4//DMRTA1//RFX7//SOX4//ZNF3//ZNF131//BHLHE41//FOSL1//TSC22D4//LCOR//RHOXF2//BHLHE40//RUNX3//CBFB//ST18//CARF//KLF7//ZNF217//TCERG1//RBBP8//MED6//CNOT7//PURG//JARID2//ARID5B//MACC1//KLF17//ZNF782//ZFP30//ZNF568//KLF3//SLC2A4RG//ZNF630//ZNF134//ZNF227//ZNF655//BLZF1//CBL//BTAF1//VGLL1//ZMYND11//BTG1//NMI//NPAT//RAP2C//SMARCD2//HSBP1//SRSF2//TBL1XR1//NCOA7// |
| GO:1990837 | sequence-specific double-stranded DNA binding | Molecular function | 50 | 776 | 582 | 17548 | 1.94273213589825 | 5.67029895674803e-06 | 0.000734870744794545 | 5.24639404307477 | 0.0859106529209622 | BAHD1//ZNF568//NFE2//PPARG//SOX4//CEBPB//ZMYND11//FOXF2//GATA2//PURG//MACC1//IRF2//JARID2//MYOG//NHLH2//NR4A2//KLF3//ZBTB4//ARID5B//RUNX3//CC2D1B//ELK3//EN1//ESR1//TFEC//IRF1//AR//MEOX2//MYB//MYBL1//NFIA//OTX2//POU3F2//RFX3//RFX4//RFX7//SMARCD2//ZNF217//BHLHE41//CARF//FOSL1//KLF7//GADD45A//ARX//PTF1A//POU4F1//SLC2A4RG//NEUROD1//NEUROG1//BHLHE40// |
| GO:0001077 | transcriptional activator activity, RNA polymerase II proximal promoter sequence-specific DNA binding | Molecular function | 24 | 279 | 582 | 17548 | 2.59365184938846 | 2.09001686297516e-05 | 0.00246241986765073 | 4.6798502098375 | 0.0412371134020619 | CEBPB//ELK3//ESR1//TFEC//PTF1A//GATA2//IRF1//AR//MEOX2//MYB//MYBL1//MYOG//NEUROD1//NFIA//NHLH2//NR4A2//OTX2//POU3F2//POU4F1//RFX4//SOX4//CARF//FOSL1//KLF7// |
| GO:0043565 | sequence-specific DNA binding | Molecular function | 64 | 1139 | 582 | 17548 | 1.6941852291001 | 2.35459495583938e-05 | 0.00254296255230653 | 4.62808379068929 | 0.109965635738832 | BAHD1//ZNF568//NFE2//PPARG//SOX4//CEBPB//ZMYND11//FOXF2//GATA2//PURG//MACC1//IRF2//JARID2//MYOG//NHLH2//NR4A2//KLF3//ZBTB4//ARID5B//RUNX3//CC2D1B//ELK3//EN1//ESR1//TFEC//IRF1//AR//MEOX2//MYB//MYBL1//NFIA//OTX2//POU3F2//RFX3//RFX4//RFX7//SMARCD2//ZNF217//BHLHE41//CARF//FOSL1//KLF7//GADD45A//ARX//PTF1A//POU4F1//SLC2A4RG//NEUROD1//NEUROG1//BHLHE40//ZBTB18//MTPN//EMX2//FOXJ3//HIVEP2//HOXA3//HOXB6//HOXC8//HOXD1//MAF//NR3C2//DMRTA1//RHOXF2B//RHOXF2// |
| GO:0000987 | proximal promoter sequence-specific DNA binding | Molecular function | 32 | 451 | 582 | 17548 | 2.13933145891909 | 4.74287900636969e-05 | 0.00472828553250394 | 4.32395795431423 | 0.0549828178694158 | CEBPB//CC2D1B//ELK3//EN1//ESR1//TFEC//GATA2//IRF1//AR//MEOX2//MYB//MYBL1//MYOG//NFIA//NHLH2//NR4A2//OTX2//POU3F2//RFX3//RFX4//RFX7//SMARCD2//ZNF217//BHLHE41//CARF//FOSL1//KLF7//PTF1A//NEUROD1//NEUROG1//BHLHE40//NFE2// |
| GO:0000978 | RNA polymerase II proximal promoter sequence-specific DNA binding | Molecular function | 31 | 436 | 582 | 17548 | 2.14377817711782 | 5.97632224428176e-05 | 0.00553236687756369 | 4.22356599333116 | 0.0532646048109966 | ESR1//PTF1A//MYOG//NEUROD1//NEUROG1//BHLHE41//BHLHE40//CEBPB//CC2D1B//ELK3//EN1//TFEC//GATA2//IRF1//AR//MEOX2//MYB//MYBL1//NFIA//NHLH2//NR4A2//OTX2//POU3F2//RFX3//RFX4//RFX7//SMARCD2//ZNF217//CARF//FOSL1//KLF7// |
| GO:0003690 | double-stranded DNA binding | Molecular function | 50 | 860 | 582 | 17548 | 1.75297690401982 | 8.34910288219788e-05 | 0.00721362489021897 | 4.07836018729993 | 0.0859106529209622 | BAHD1//ZNF568//NFE2//PPARG//SOX4//CEBPB//ZMYND11//FOXF2//GATA2//PURG//MACC1//IRF2//JARID2//MYOG//NHLH2//NR4A2//KLF3//ZBTB4//ARID5B//RUNX3//CC2D1B//ELK3//EN1//ESR1//TFEC//IRF1//AR//MEOX2//MYB//MYBL1//NFIA//OTX2//POU3F2//RFX3//RFX4//RFX7//SMARCD2//ZNF217//BHLHE41//CARF//FOSL1//KLF7//GADD45A//ARX//PTF1A//POU4F1//SLC2A4RG//NEUROD1//NEUROG1//BHLHE40// |
| GO:0001228 | transcriptional activator activity, RNA polymerase II transcription regulatory region sequence-specific DNA binding | Molecular function | 29 | 417 | 582 | 17548 | 2.09684623435272 | 0.000150514969473752 | 0.0121917125273739 | 3.82242030514289 | 0.0498281786941581 | CEBPB//ELK3//ESR1//TFEC//PTF1A//GATA2//IRF1//AR//MEOX2//MYB//MYBL1//MYOG//NEUROD1//NFIA//NHLH2//NR4A2//OTX2//POU3F2//POU4F1//RFX4//SOX4//CARF//FOSL1//KLF7//FOXJ3//FOXF2//MACC1//IRF2//MAF// |
| GO:0003677 | DNA binding | Molecular function | 115 | 2520 | 582 | 17548 | 1.37594774450445 | 0.000178009656339533 | 0.0135706185068256 | 3.74955643824601 | 0.197594501718213 | BAHD1//ZNF568//NFE2//PPARG//SOX4//CEBPB//ZMYND11//FOXF2//GATA2//PURG//MACC1//IRF2//JARID2//MYOG//NHLH2//NR4A2//KLF3//ZBTB4//ARID5B//RUNX3//CC2D1B//ELK3//EN1//ESR1//TFEC//IRF1//AR//MEOX2//MYB//MYBL1//NFIA//OTX2//POU3F2//RFX3//RFX4//RFX7//SMARCD2//ZNF217//BHLHE41//CARF//FOSL1//KLF7//GADD45A//ARX//PTF1A//POU4F1//ST18//SLC2A4RG//RBBP8//UNG//NABP1//TRERF1//H3F3C//ZBTB18//MTPN//EMX2//FOXJ3//HIVEP2//HOXA3//HOXB6//HOXC8//HOXD1//MAF//NR3C2//NEUROD1//DMRTA1//RHOXF2B//RHOXF2//KLF17//RCOR1//ARID4B//TNF//TBL1XR1//NEUROG1//BHLHE40//TSHZ1//ZNF268//KLF12//ZNF782//ZNF800//ZFP30//KDM2A//MYT1L//ZFPM2//SPO11//HBP1//KIF4B//AGFG1//ZNF680//IMPDH1//SYCP3//POLR2K//ZFAND6//ATR//TET2//STRBP//WRNIP1//ZNF630//ZBTB26//MIER1//ATXN1//SOS2//SYCP1//ZNF3//ZNF131//ZNF134//ZNF208//ZNF227//ZNF655//LCOR//ZIC5//BLZF1//CBFB//BTAF1//DTD1// |
| GO:0005515 | protein binding | Molecular function | 423 | 11565 | 582 | 17548 | 1.10280663751722 | 0.000216895774195333 | 0.015616495742064 | 3.66374890931142 | 0.72680412371134 | STX6//STX12//ZFPM2//GATA2//AR//ESR1//NEUROD1//NHLH2//BHLHE41//BHLHE40//TCERG1//RBBP8//CRKL//SAMSN1//YES1//CBL//C5AR1//LDLR//TNF//CASP3//RNF125//ZMAT3//CLASP2//IQGAP2//KLHL3//PXK//ENAH//TMOD1//MED28//PHACTR2//COBLL1//ARHGAP1//NCK1//SH3BGR//SH3BGRL//SRSF2//RAPGEF4//RAB3GAP1//ARHGEF4//RALGPS2//SOS2//LAMTOR3//PSD//CSF2RA//EREG//RASGEF1A//IL2//IL5//MET//BTC//RAPGEF2//STRAP//ADCYAP1//CADM2//GJA1//LRP6//NTF3//CCL2//BMP3//WNT7A//FLRT1//NPTN//DLL1//MYD88//EDN1//IFNA1//IFNG//INHBB//IL36G//AIMP1//SNX2//SOCS5//IGF1//SIVA1//EFNA5//CD2AP//SPRED1//NPNT//C1QTNF9//CALCB//SNAP25//NOS1//PPP3CB//CCL28//CCL8//NR4A2//SKP1//TBL1XR1//DLG5//CETN1//DCX//DPYSL2//FMR1//KIF4B//SYBU//CLIP1//KIF13A//NPAT//HPCAL4//PPARG//PRKAA1//MID1IP1//ATXN1//MACC1//OGN//VEGFB//DYNLL2//MED6//AGTR2//FOXF2//RCOR1//CNOT7//MDFIC//JARID2//NFIA//TRERF1//AKAP11//CALM2//AKAP6//OTX2//STMN2//VBP1//SPHK2//DENND1B//SBF2//EPS15//PTPN22//OSTF1//ADAM12//QKI//EVI5//USP6NL//APCDD1//FRZB//FZD3//FZD6//BMPR2//LRP8//UBE4B//CDK19//CNTNAP2//GSTM3//MDM4//EGLN1//BTG1//BLZF1//CEBPB//GADD45A//DLG2//CCNY//MAP3K1//ZBTB4//CD8A//MTMR9//CDC27//DDX6//ARFIP1//PRKAR2B//TMED10//SYT6//PLP2//CCR1//G3BP2//GRIK2//CDK6//SOD1//ARAP2//APPL1//AGFG1//PEX5L//EIF3B//TGFBR2//RAB6B//ZFAND6//DCUN1D4//RNF180//LONRF3//LONRF1//SPOPL//UBE2W//UBE2G1//ARRDC3//TSC1//NRXN1//ACBD3//SNX5//ZMYND11//TACC2//NCOA7//IL20RA//ANP32B//ATAD2//TNKS//CLCN5//ARL6IP1//HSPB8//GJB2//HPRT1//HSBP1//ST8SIA3//RAB9B//POU3F2//ATG16L1//WRNIP1//ZBTB26//PTPRG//BNIP3//ZNF3//DCTPP1//TRIM8//CD84//NMI//SLIT2//CALCOCO2//GPD1//RBM44//FIBIN//MGST1//NEUROG1//CHMP3//BCL2A1//DMRTA1//GNPTG//ACVR1//CD69//MIER1//FAM110C//MZT1//UBXN2B//PLAA//SCAF8//PIK3CB//CCT6A//MB21D2//TES//PAK6//DYNC1LI2//RAET1E//H3F3C//MYOG//RRAGD//SOX4//TFEC//PTF1A//GLRX//NFE2//TBCA//PMEPA1//AKAP4//POU4F1//PTGES3//DNAJB4//RP2//LDLRAD4//HOXA3//DSC2//TREM1//CHRM2//IL1RAP//GPHN//RTN3//ZBTB18//PROCR//DCTN6//RAI2//FASTK//EGLN3//KLF12//ERP27//SLAIN1//FRMD6//MIEF2//KLF17//TMEM182//VPS37A//RAD9B//PDIK1L//SH3D19//PATE1//C12ORF50//DCP2//DOK1//CC2D1B//ELK3//HTR3D//RAB12//EMX2//SMIM14//HS3ST5//CPEB3//BAHD1//WDR47//MMRN1//SV2C//KDM2A//WDFY3//FBXO28//OTUD3//TTC33//SPO11//CNOT6L//HEPACAM2//LCLAT1//MSRB3//PAN3//NUTM1//INTS7//RAI14//HBP1//GLRB//FAM126B//FAM133A//GSTA2//ST8SIA5//LRP12//NRBF2//HOXB6//FAM92B//ANKRD45//IRF1//IRF2//KCNA4//NHLRC2//KCNJ3//KCNJ10//KCNS2//KLC1//FAM19A1//MAF//MBNL1//MDH1//MEOX2//MEP1A//MLLT6//NR3C2//MTM1//MTRR//MYB//NEU1//DCAF8//MEMO1//COMMD2//KLF3//PRR16//YTHDF2//RASD1//ARID4B//PIGA//ATR//TET2//PAQR5//SMU1//CCDC25//RBM41//DRAM1//STRBP//PPP3R2//DBNDD2//TEX11//FSTL5//MAP3K7CL//TMEM159//CNOT6//HECW2//STIM2//USP28//RBM25//MPP4//RFX3//RFX4//RRM2//RTN1//SMOC1//METTL17//NABP1//SLC1A1//KRTAP4-11//SMARCD2//SNRPN//BTF3//TRPC3//UNG//CA1//ZNF217//ZNF227//PTP4A1//ZNF655//CENPO//PLEKHF2//SNIP1//MMRN2//UBA5//ELOVL7//FAM192A//C16ORF70//FOSL1//TSC22D4//CAMLG//ARID5B//PCGF5//LCOR//RHOXF2//KBTBD8//TMEM185A//PPP1R15B//FAM104A//CDC14A//RUNX3//CBFB//SLC4A4//C5ORF30//DIRAS3//SESTD1//BTF3L4//WDR20//RPS6KA5//SYCE1//PIGS//ITM2A//PHF14//ULK2//SECISBP2L//EFCAB14// |
| GO:0070851 | growth factor receptor binding | Molecular function | 13 | 133 | 582 | 17548 | 2.94711004314911 | 0.000482521775919994 | 0.0329130642943322 | 3.31648308242694 | 0.0223367697594502 | FLRT1//NPTN//IL2//IL5//IL36G//EREG//SNX2//BTC//YES1//CBL//SOCS5//CD2AP//VEGFB// |
| GO:0005154 | epidermal growth factor receptor binding | Molecular function | 6 | 33 | 582 | 17548 | 5.48203686348016 | 0.000669114842890186 | 0.0416101084443451 | 3.17449933611399 | 0.0103092783505155 | EREG//SNX2//BTC//YES1//CBL//SOCS5// |
| GO:0004535 | poly(A)-specific ribonuclease activity | Molecular function | 4 | 13 | 582 | 17548 | 9.27729315358181 | 0.000674237868311148 | 0.0416101084443451 | 3.17118685914711 | 0.00687285223367698 | CNOT6L//PAN3//CNOT7//CNOT6// |
| GO:0031624 | ubiquitin conjugating enzyme binding | Molecular function | 6 | 34 | 582 | 17548 | 5.32080048514251 | 0.000789999275418047 | 0.0465381391337177 | 3.10237330704132 | 0.0103092783505155 | DCUN1D4//RNF180//GRIK2//RNF125//LONRF3//LONRF1// |
| GO:0005041 | low-density lipoprotein particle receptor activity | Molecular function | 4 | 14 | 582 | 17548 | 8.6146293568974 | 0.000919302243992058 | 0.0498361913082975 | 3.03654167978085 | 0.00687285223367698 | LRP12//LDLR//LRP6//LRP8// |
| GO:0017124 | SH3 domain binding | Molecular function | 12 | 125 | 582 | 17548 | 2.89451546391753 | 0.000922892431635138 | 0.0498361913082975 | 3.03484891551765 | 0.0206185567010309 | EPS15//CD2AP//PTPN22//OSTF1//GJA1//ARHGAP1//ENAH//SH3BGR//SH3BGRL//ADAM12//CBL//QKI// |
| GO:0005126 | cytokine receptor binding | Molecular function | 20 | 278 | 582 | 17548 | 2.16915127691661 | 0.00102144929205237 | 0.0529519312999949 | 2.99078318823707 | 0.0343642611683849 | MYD88//CASP3//IFNA1//IFNG//IL2//IL5//IL36G//INHBB//BMP3//SIVA1//TNF//NTF3//EFNA5//CD2AP//SPRED1//CCL28//CCL2//CCL8//TGFBR2//VEGFB// |
| GO:0070888 | E-box binding | Molecular function | 6 | 37 | 582 | 17548 | 4.88938422959041 | 0.0012554018249716 | 0.0625769525062767 | 2.90121724435305 | 0.0103092783505155 | PTF1A//MYOG//NEUROD1//NEUROG1//BHLHE41//BHLHE40// |
| GO:0000989 | transcription factor activity, transcription factor binding | Molecular function | 36 | 643 | 582 | 17548 | 1.68809222234692 | 0.00158676073518573 | 0.076164515288915 | 2.7994885547968 | 0.0618556701030928 | PPARG//TCERG1//TRERF1//ZFPM2//NEUROD1//POU3F2//SOX4//RBBP8//MED6//MYB//MYBL1//CNOT7//BHLHE41//BHLHE40//ZMYND11//BTG1//CBFB//NMI//ZBTB18//TFEC//NFE2//NPAT//VGLL1//POU4F1//RAP2C//SMARCD2//ARID5B//KLF7//KLF12//ELK3//RCOR1//HSBP1//SRSF2//TBL1XR1//NCOA7//NEUROG1// |
| GO:0000988 | transcription factor activity, protein binding | Molecular function | 36 | 648 | 582 | 17548 | 1.67506681939672 | 0.00181013827339733 | 0.0825923133926503 | 2.74228824884364 | 0.0618556701030928 | NEUROG1//PPARG//TCERG1//TRERF1//ZFPM2//NEUROD1//POU3F2//SOX4//RBBP8//MED6//MYB//MYBL1//CNOT7//BHLHE41//BHLHE40//ZMYND11//BTG1//CBFB//NMI//ZBTB18//TFEC//NFE2//NPAT//VGLL1//POU4F1//RAP2C//SMARCD2//ARID5B//KLF7//KLF12//ELK3//RCOR1//HSBP1//SRSF2//TBL1XR1//NCOA7// |
| GO:0044390 | ubiquitin-like protein conjugating enzyme binding | Molecular function | 6 | 40 | 582 | 17548 | 4.52268041237113 | 0.00190584617564731 | 0.0825923133926503 | 2.71991215499017 | 0.0103092783505155 | DCUN1D4//RNF180//GRIK2//RNF125//LONRF3//LONRF1// |
| GO:0003705 | transcription factor activity, RNA polymerase II distal enhancer sequence-specific binding | Molecular function | 10 | 101 | 582 | 17548 | 2.98526759892484 | 0.00191185910631135 | 0.0825923133926503 | 2.71854411603802 | 0.0171821305841924 | MEOX2//POU4F1//ARX//CEBPB//FOXF2//MYOG//VGLL1//RFX3//BHLHE41//BHLHE40// |
| GO:0030228 | lipoprotein particle receptor activity | Molecular function | 4 | 17 | 582 | 17548 | 7.09440064685668 | 0.00201945189543168 | 0.0844261179509502 | 2.6947664875286 | 0.00687285223367698 | LRP12//LDLR//LRP6//LRP8// |
| GO:0003712 | transcription cofactor activity | Molecular function | 32 | 566 | 582 | 17548 | 1.7046616395274 | 0.00243444468216073 | 0.0985950096275096 | 2.61360008947105 | 0.0549828178694158 | TCERG1//TRERF1//ZFPM2//NEUROD1//POU3F2//SOX4//RBBP8//MED6//ZBTB18//TFEC//NFE2//NPAT//VGLL1//POU4F1//RAP2C//SMARCD2//ARID5B//KLF7//CBFB//ZMYND11//KLF12//ELK3//RCOR1//HSBP1//SRSF2//BHLHE41//TBL1XR1//BHLHE40//NCOA7//PPARG//BTG1//NMI// |
| GO:0017147 | Wnt-protein binding | Molecular function | 5 | 31 | 582 | 17548 | 4.86309721760337 | 0.00327955870937812 | 0.128797214768304 | 2.4841845901223 | 0.00859106529209622 | APCDD1//FRZB//LRP6//FZD3//FZD6// |
| GO:0016896 | exoribonuclease activity, producing 5'-phosphomonoesters | Molecular function | 5 | 33 | 582 | 17548 | 4.56836405290014 | 0.00433859636061199 | 0.165377084804504 | 2.36265075238589 | 0.00859106529209622 | CNOT7//DCP2//CNOT6L//PAN3//CNOT6// |
| GO:0003713 | transcription coactivator activity | Molecular function | 20 | 317 | 582 | 17548 | 1.9022840851193 | 0.00468981693644792 | 0.168976275857839 | 2.32884410931949 | 0.0343642611683849 | ZFPM2//NEUROD1//POU3F2//SOX4//MED6//NCOA7//PPARG//TRERF1//ZBTB18//TCERG1//TFEC//NFE2//NPAT//VGLL1//POU4F1//RAP2C//SMARCD2//ARID5B//KLF7//CBFB// |
| GO:0001076 | transcription factor activity, RNA polymerase II transcription factor binding | Molecular function | 14 | 191 | 582 | 17548 | 2.21003580360195 | 0.00469378544049554 | 0.168976275857839 | 2.32847676649255 | 0.0240549828178694 | TCERG1//TRERF1//ZFPM2//NEUROD1//POU3F2//SOX4//RBBP8//MED6//MYB//MYBL1//CNOT7//BHLHE41//BHLHE40//PPARG// |
| GO:0016004 | phospholipase activator activity | Molecular function | 3 | 11 | 582 | 17548 | 8.22305529522024 | 0.0049072930822728 | 0.171887887422312 | 2.30915800352045 | 0.00515463917525773 | CASP3//PLAA//CCL8// |
| GO:0005488 | binding | Molecular function | 519 | 15006 | 582 | 17548 | 1.04281448932455 | 0.00506968387376372 | 0.172902902642047 | 2.2950191207772 | 0.891752577319588 | AIMP1//DTD1//ACBD3//ACBD5//STX6//STX12//HPRT1//PEX5L//DCTPP1//RPS6KA5//CPEB2//CPEB3//CPEB1//BAHD1//ZNF568//NFE2//PPARG//SOX4//CEBPB//ZMYND11//FOXF2//GATA2//PURG//MACC1//IRF2//JARID2//MYOG//NHLH2//NR4A2//KLF3//ZBTB4//ARID5B//RUNX3//CC2D1B//ELK3//EN1//ESR1//TFEC//IRF1//AR//MEOX2//MYB//MYBL1//NFIA//OTX2//POU3F2//RFX3//RFX4//RFX7//SMARCD2//ZNF217//BHLHE41//CARF//FOSL1//KLF7//GADD45A//ARX//PTF1A//POU4F1//ST18//MBNL1//ZFPM2//NEUROD1//BHLHE40//TCERG1//RBBP8//SLC2A4RG//LDLR//ITM2A//CRKL//SAMSN1//YES1//CBL//SYT6//C5AR1//TNF//CASP3//RNF125//ZMAT3//FMR1//CLASP2//IMPDH1//KCMF1//TSHZ1//ZBTB18//ZNF268//KLF12//ZNF782//ZNF800//ZFP30//KDM2A//MYT1L//SPO11//HBP1//KIF4B//HIVEP2//AGFG1//ZNF680//NEUROG1//SYCP3//POLR2K//ZFAND6//ATR//TET2//STRBP//WRNIP1//ZNF630//ZBTB26//MIER1//ATXN1//SOS2//SYCP1//ZNF3//ZNF131//ZNF134//ZNF208//ZNF227//ZNF655//LCOR//ZIC5//BLZF1//CBFB//BTAF1//ATAD2//PRKAA1//GMNC//UNG//NABP1//FASTK//STRAP//PABPC5//DDX6//ELAVL4//SCAF8//PAN3//FAM133B//TES//CNOT7//HOXB6//RBM44//YTHDF2//CNOT6//RBM25//SRSF2//SNRPN//BTF3//TBCA//SNIP1//RTCA//EIF3B//CCT6A//QKI//SECISBP2L//G3BP2//IQGAP2//KLHL3//PXK//ENAH//TMOD1//MED28//PHACTR2//COBLL1//ARHGAP1//NCK1//SH3BGR//SH3BGRL//RAPGEF4//RAB3GAP1//ARHGEF4//RALGPS2//LAMTOR3//PSD//CSF2RA//EREG//RASGEF1A//IL2//IL5//MET//BTC//RAPGEF2//ADCYAP1//CADM2//GJA1//LRP6//NTF3//CCL2//BMP3//WNT7A//FLRT1//NPTN//DLL1//MYD88//EDN1//IFNA1//IFNG//INHBB//IL36G//SNX2//SOCS5//IGF1//SIVA1//EFNA5//CD2AP//SPRED1//NPNT//C1QTNF9//CALCB//SNAP25//HSD11B1//NR3C2//PAQR5//ADH4//EGLN3//NOS1//EGLN1//MSMO1//SOD1//CETN1//DSC2//EPS15//MMRN1//SULF1//GPD2//MICU3//MAN1A1//HPCAL4//PPP3CB//PPP3R2//PCDHB10//FSTL5//STIM2//SMOC1//SMOC2//SPOCK1//LRP8//PRRG4//CALM2//PLA2G12A//SLIT2//NRXN1//CD69//EFCAB14//MED6//CDK6//CALCOCO2//GPHN//RTN3//ANP32B//PROCR//DCTN6//PTGES3//RAI2//TMED10//STMN2//DNAJB4//AKAP11//CLCN5//ERP27//SLAIN1//FRMD6//CCR1//MIEF2//KLF17//TMEM182//NCOA7//RAET1E//VPS37A//UBXN2B//DYNLL2//RAD9B//APCDD1//PDIK1L//SH3D19//PATE1//C12ORF50//DCX//DCP2//DLG2//DOK1//DPYSL2//AGTR2//HTR3D//RAB12//EMX2//SMIM14//CCNY//HS3ST5//WDR47//SV2C//WDFY3//DCUN1D4//RCOR1//ARL6IP1//FBXO28//OTUD3//TTC33//CNOT6L//HEPACAM2//LCLAT1//MSRB3//NUTM1//INTS7//CNTNAP2//APPL1//RAI14//PTPN22//HSPB8//OSTF1//SNX5//GLRB//FAM126B//FAM133A//GSTA2//GSTM3//ST8SIA5//LRP12//MDFIC//NRBF2//HOXA3//HSBP1//FAM92B//ANKRD45//SPOPL//KCNA4//NHLRC2//KCNJ3//KCNJ10//KCNS2//KLC1//FAM19A1//MAF//MDH1//MDM4//MAP3K1//MEP1A//MGST1//MLLT6//H3F3C//MZT1//MTM1//MTRR//NEU1//NPAT//OGN//DCAF8//MEMO1//COMMD2//RAB9B//PRR16//RAB6B//CHMP3//RASD1//ARID4B//PIGA//PIK3CB//PLP2//IL20RA//ATG16L1//SMU1//CCDC25//UBE2W//RBM41//DRAM1//SYBU//PRKAR2B//DBNDD2//TEX11//CCL28//SPHK2//MAP3K7CL//PAK6//PMEPA1//TMEM159//HECW2//ARRDC3//USP28//PTPRG//MID1IP1//RRAGD//MPP4//BCL2A1//RP2//RRM2//CLIP1//RTN1//KIF13A//FAM110C//METTL17//SKP1//SLC1A1//KRTAP4-11//BMPR2//MTMR9//BNIP3//BTG1//TGFBR2//TRPC3//TSC1//VBP1//VEGFB//CA1//PTP4A1//EVI5//CENPO//PLEKHF2//TBL1XR1//FZD3//MMRN2//LONRF3//UBA5//ELOVL7//FAM192A//C16ORF70//ADAM12//TRIM8//TSC22D4//SBF2//CAMLG//FZD6//PCGF5//RHOXF2//KBTBD8//TMEM185A//PPP1R15B//FAM104A//CDC14A//TNKS//SLC4A4//CD84//ACVR1//C5ORF30//DIRAS3//NMI//SESTD1//BTF3L4//LONRF1//WDR20//DLG5//CD8A//SYCE1//PLAA//PIGS//AKAP6//PHF14//ULK2//CDC27//UBE4B//CLCN6//DGKH//DYNC1LI2//AK4//ACSL1//CDK19//CMPK1//STK33//UBE2G1//DGKE//ARL5A//RAB30//GLUD2//ARL15//RAP2C//RERGL//RAB28//HAPLN1//ARFIP1//ARAP2//OR13A1//CCL8//TRERF1//GPR183//CHRM2//RNF145//XAF1//SLC30A5//DENND1B//USP6NL//FRZB//NPEPL1//GRIK2//RNF180//ST8SIA3//TNFAIP8L3//TACC2//NLN//THOP1//GJB2//GPD1//FIBIN//DMRTA1//GNPTG//REG3G//MTPN//FOXJ3//HOXC8//HOXD1//RHOXF2B//MB21D2//RNF38//ARSK//ESCO2//ZNRF2//ENPP4//DDHD2//STEAP2//NOB1//IDS//RNF186//CPPED1//CACNA2D3//ENPP5//LMLN//GLRX//VKORC1L1//AKAP4//SLC2A5//LDLRAD4//TREM1//IL1RAP// |
| GO:0042813 | Wnt-activated receptor activity | Molecular function | 4 | 22 | 582 | 17548 | 5.48203686348016 | 0.00544334578896008 | 0.180886567756212 | 2.26413407621623 | 0.00687285223367698 | FRZB//LRP6//FZD3//FZD6// |
| GO:0004532 | exoribonuclease activity | Molecular function | 5 | 35 | 582 | 17548 | 4.3073146784487 | 0.00562098322641925 | 0.182119856535984 | 2.25018771068366 | 0.00859106529209622 | CNOT7//DCP2//CNOT6L//PAN3//CNOT6// |
| GO:0005246 | calcium channel regulator activity | Molecular function | 5 | 36 | 582 | 17548 | 4.18766704849179 | 0.00635312325229697 | 0.196970103236954 | 2.19701271915345 | 0.00859106529209622 | CALM2//CRISP1//HPCAL4//STIM2//NRXN1// |
| GO:0035925 | mRNA 3'-UTR AU-rich region binding | Molecular function | 3 | 12 | 582 | 17548 | 7.53780068728522 | 0.00638329038267906 | 0.196970103236954 | 2.19495539860221 | 0.00515463917525773 | CPEB2//CPEB3//CPEB1// |
| GO:0008146 | sulfotransferase activity | Molecular function | 6 | 52 | 582 | 17548 | 3.47898493259318 | 0.00723805413765812 | 0.218151585172208 | 2.14037817287216 | 0.0103092783505155 | CHST7//CHST1//SULT6B1//HS3ST5//NDST3//DSEL// |
| GO:0061630 | ubiquitin protein ligase activity | Molecular function | 15 | 224 | 582 | 17548 | 2.01905375552283 | 0.00794796012163611 | 0.233164976745295 | 2.09974432060058 | 0.0257731958762887 | UBE4B//RNF38//RNF145//ZNRF2//RNF180//MDM4//RNF125//UBE2W//HECW2//SKP1//UBE2G1//LONRF3//CBL//UBE3B//LONRF1// |
| GO:0060229 | lipase activator activity | Molecular function | 3 | 13 | 582 | 17548 | 6.95796986518636 | 0.00809600613698942 | 0.233164976745295 | 2.09172917129731 | 0.00515463917525773 | CCL8//CASP3//PLAA// |
| GO:0017091 | AU-rich element binding | Molecular function | 4 | 25 | 582 | 17548 | 4.82419243986254 | 0.00870377154937105 | 0.240001870808189 | 2.06029251656014 | 0.00687285223367698 | CPEB2//CPEB3//CPEB1//ELAVL4// |
| GO:0030371 | translation repressor activity | Molecular function | 4 | 25 | 582 | 17548 | 4.82419243986254 | 0.00870377154937105 | 0.240001870808189 | 2.06029251656014 | 0.00687285223367698 | CPEB2//CPEB3//CPEB1//FMR1// |
| GO:0061659 | ubiquitin-like protein ligase activity | Molecular function | 15 | 229 | 582 | 17548 | 1.97496961238914 | 0.00963285363342139 | 0.255471815017638 | 2.01624503855951 | 0.0257731958762887 | UBE4B//RNF38//RNF145//ZNRF2//RNF180//MDM4//RNF125//UBE2W//HECW2//SKP1//UBE2G1//LONRF3//CBL//UBE3B//LONRF1// |
| GO:0030545 | receptor regulator activity | Molecular function | 27 | 502 | 582 | 17548 | 1.62167823551156 | 0.00967921002182967 | 0.255471815017638 | 2.01416008661313 | 0.0463917525773196 | EDN1//IFNA1//IFNG//IL2//IL5//INHBB//IL36G//BMP3//TNF//WNT7A//AIMP1//C1QTNF9//IGF1//ADCYAP1//CALCB//CCL28//CCL2//CCL8//EREG//MACC1//NTF3//OGN//BTC//VEGFB//EFNA5//PATE1//AGTR2// |
| GO:0000900 | translation repressor activity, mRNA regulatory element binding | Molecular function | 3 | 14 | 582 | 17548 | 6.46097201767305 | 0.0100532890168978 | 0.255471815017638 | 1.9976918319542 | 0.00515463917525773 | CPEB2//CPEB3//CPEB1// |
| GO:0005095 | GTPase inhibitor activity | Molecular function | 3 | 14 | 582 | 17548 | 6.46097201767305 | 0.0100532890168978 | 0.255471815017638 | 1.9976918319542 | 0.00515463917525773 | IQGAP2//CPEB2//SLIT2// |
| GO:0003714 | transcription corepressor activity | Molecular function | 15 | 232 | 582 | 17548 | 1.94943121222894 | 0.0107719119139906 | 0.268469189240996 | 1.96770720663671 | 0.0257731958762887 | TCERG1//RBBP8//ZMYND11//KLF12//ELK3//TFEC//RCOR1//ZFPM2//HSBP1//NPAT//POU4F1//SRSF2//BHLHE41//TBL1XR1//BHLHE40// |
| GO:0001227 | transcriptional repressor activity, RNA polymerase II transcription regulatory region sequence-specific DNA binding | Molecular function | 15 | 233 | 582 | 17548 | 1.94106455466572 | 0.011174265534916 | 0.273242417608512 | 1.95178101268065 | 0.0257731958762887 | TSHZ1//CEBPB//CC2D1B//EN1//MYT1L//RCOR1//ZFPM2//ZNF217//BHLHE41//BHLHE40//ARX//PURG//JARID2//ZBTB4//ARID5B// |
| GO:0003707 | steroid hormone receptor activity | Molecular function | 6 | 59 | 582 | 17548 | 3.06622400838721 | 0.0132014196529434 | 0.316834071670642 | 1.87937936316633 | 0.0103092783505155 | AR//ESR1//NR3C2//NR4A2//PPARG//PAQR5// |
| GO:0051020 | GTPase binding | Molecular function | 33 | 665 | 582 | 17548 | 1.49622509882955 | 0.0141340219225087 | 0.33304895293766 | 1.84973423941035 | 0.0567010309278351 | RAPGEF4//RAB3GAP1//ARHGEF4//RALGPS2//SOS2//LAMTOR3//PSD//CSF2RA//EREG//RASGEF1A//IL2//IL5//MET//BTC//RAPGEF2//SPHK2//IQGAP2//DENND1B//SBF2//ARHGAP1//EVI5//USP6NL//ARAP2//APPL1//AGFG1//PEX5L//PAK6//SOD1//POU4F1//ATG16L1//RRAGD//BNIP3//AIMP1// |
| GO:0001105 | RNA polymerase II transcription coactivator activity | Molecular function | 5 | 44 | 582 | 17548 | 3.4262730396751 | 0.0147491402995844 | 0.33796545555669 | 1.83123329317922 | 0.00859106529209622 | MED6//ZFPM2//NEUROD1//POU3F2//SOX4// |
| GO:0048018 | receptor ligand activity | Molecular function | 25 | 472 | 582 | 17548 | 1.596991671035 | 0.014864221424947 | 0.33796545555669 | 1.82785783383238 | 0.0429553264604811 | EDN1//IFNA1//IFNG//IL2//IL5//INHBB//IL36G//BMP3//TNF//WNT7A//AIMP1//C1QTNF9//IGF1//ADCYAP1//CALCB//CCL28//CCL2//CCL8//EREG//MACC1//NTF3//OGN//BTC//VEGFB//EFNA5// |
| GO:0005125 | cytokine activity | Molecular function | 14 | 220 | 582 | 17548 | 1.91871290221806 | 0.0152854679545072 | 0.341551146017954 | 1.81572126109561 | 0.0240549828178694 | CCL28//CCL2//CCL8//EDN1//IFNA1//IFNG//IL2//IL5//INHBB//IL36G//BMP3//TNF//WNT7A//AIMP1// |
| GO:0031369 | translation initiation factor binding | Molecular function | 4 | 30 | 582 | 17548 | 4.02016036655212 | 0.0165580872830933 | 0.36371662913371 | 1.78098983242883 | 0.00687285223367698 | OTX2//NCK1//FMR1//EIF3B// |
| GO:0003951 | NAD+ kinase activity | Molecular function | 3 | 17 | 582 | 17548 | 5.32080048514251 | 0.017447669134025 | 0.364712567704781 | 1.75826258302253 | 0.00515463917525773 | DGKH//SPHK2//DGKE// |
| GO:0004675 | transmembrane receptor protein serine/threonine kinase activity | Molecular function | 3 | 17 | 582 | 17548 | 5.32080048514251 | 0.017447669134025 | 0.364712567704781 | 1.75826258302253 | 0.00515463917525773 | TGFBR2//ACVR1//BMPR2// |
| GO:0008484 | sulfuric ester hydrolase activity | Molecular function | 3 | 17 | 582 | 17548 | 5.32080048514251 | 0.017447669134025 | 0.364712567704781 | 1.75826258302253 | 0.00515463917525773 | ARSK//SULF1//IDS// |
| GO:0000175 | 3'-5'-exoribonuclease activity | Molecular function | 4 | 31 | 582 | 17548 | 3.8904777740827 | 0.0185252767014215 | 0.370296625345506 | 1.73223529649287 | 0.00687285223367698 | CNOT6L//PAN3//CNOT7//CNOT6// |
| GO:0050699 | WW domain binding | Molecular function | 4 | 31 | 582 | 17548 | 3.8904777740827 | 0.0185252767014215 | 0.370296625345506 | 1.73223529649287 | 0.00687285223367698 | NFE2//ENAH//PMEPA1//RAPGEF2// |
| GO:0008013 | beta-catenin binding | Molecular function | 7 | 82 | 582 | 17548 | 2.57388316151203 | 0.0190097894379291 | 0.370296625345506 | 1.7210226935739 | 0.0120274914089347 | ESR1//CD2AP//AR//NR4A2//SKP1//TBL1XR1//DLG5// |
| GO:0001078 | transcriptional repressor activity, RNA polymerase II proximal promoter sequence-specific DNA binding | Molecular function | 10 | 141 | 582 | 17548 | 2.13838317369794 | 0.0191434212177075 | 0.370296625345506 | 1.71798044465766 | 0.0171821305841924 | TSHZ1//CEBPB//CC2D1B//EN1//MYT1L//RCOR1//ZFPM2//ZNF217//BHLHE41//BHLHE40// |
| GO:0001085 | RNA polymerase II transcription factor binding | Molecular function | 10 | 141 | 582 | 17548 | 2.13838317369794 | 0.0191434212177075 | 0.370296625345506 | 1.71798044465766 | 0.0171821305841924 | ESR1//NEUROD1//NHLH2//BHLHE41//BHLHE40//TCERG1//RBBP8//ZFPM2//GATA2//AR// |
| GO:0008083 | growth factor activity | Molecular function | 11 | 163 | 582 | 17548 | 2.03474374380705 | 0.020141250423159 | 0.383868537476677 | 1.69591357077244 | 0.0189003436426117 | EREG//MACC1//IGF1//IL2//IL5//INHBB//NTF3//OGN//BMP3//BTC//VEGFB// |
| GO:0001104 | RNA polymerase II transcription cofactor activity | Molecular function | 8 | 103 | 582 | 17548 | 2.34184099022454 | 0.0211874190888272 | 0.397955002016233 | 1.67392194294691 | 0.013745704467354 | ZFPM2//NEUROD1//POU3F2//SOX4//TCERG1//RBBP8//MED6//TRERF1// |
| GO:0003730 | mRNA 3'-UTR binding | Molecular function | 6 | 66 | 582 | 17548 | 2.74101843174008 | 0.0219615304456386 | 0.406602049393538 | 1.65833739824127 | 0.0103092783505155 | CPEB2//CPEB3//CPEB1//ELAVL4//FMR1//SECISBP2L// |
| GO:0001191 | transcriptional repressor activity, RNA polymerase II transcription factor binding | Molecular function | 5 | 50 | 582 | 17548 | 3.01512027491409 | 0.024502524857632 | 0.441045447437376 | 1.61078916154407 | 0.00859106529209622 | TCERG1//RBBP8//CNOT7//BHLHE41//BHLHE40// |
| GO:0051018 | protein kinase A binding | Molecular function | 5 | 50 | 582 | 17548 | 3.01512027491409 | 0.024502524857632 | 0.441045447437376 | 1.61078916154407 | 0.00859106529209622 | PRKAR2B//ACBD3//AKAP6//AKAP11//AKAP4// |
| GO:0008408 | 3'-5' exonuclease activity | Molecular function | 5 | 51 | 582 | 17548 | 2.95600026952362 | 0.0264503118687122 | 0.463237894349338 | 1.57756920294591 | 0.00859106529209622 | CNOT7//CNOT6L//PAN3//CNOT6//RAD9B// |
| GO:0016796 | exonuclease activity, active with either ribo- or deoxyribonucleic acids and producing 5'-phosphomonoesters | Molecular function | 5 | 51 | 582 | 17548 | 2.95600026952362 | 0.0264503118687122 | 0.463237894349338 | 1.57756920294591 | 0.00859106529209622 | CNOT7//DCP2//CNOT6L//PAN3//CNOT6// |
| GO:0001784 | phosphotyrosine residue binding | Molecular function | 4 | 35 | 582 | 17548 | 3.44585174275896 | 0.0278043193392159 | 0.480458638181651 | 1.55588773215841 | 0.00687285223367698 | CRKL//SAMSN1//YES1//CBL// |
| GO:0016782 | transferase activity, transferring sulfur-containing groups | Molecular function | 6 | 71 | 582 | 17548 | 2.54798896471613 | 0.0301930075062701 | 0.514870233264817 | 1.52009362502516 | 0.0103092783505155 | CHST7//CHST1//SULT6B1//DSEL//HS3ST5//NDST3// |
| GO:0001158 | enhancer sequence-specific DNA binding | Molecular function | 7 | 94 | 582 | 17548 | 2.24530233238283 | 0.0366472208249141 | 0.60757418565272 | 1.43595895488831 | 0.0120274914089347 | ARX//PTF1A//GATA2//MEOX2//POU4F1//SMARCD2//SLC2A4RG// |
| GO:0098772 | molecular function regulator | Molecular function | 75 | 1848 | 582 | 17548 | 1.22366894274111 | 0.037587614296673 | 0.60757418565272 | 1.42495523828381 | 0.128865979381443 | DNAJB4//FLRT1//NCK1//SOCS5//CASP3//PRKAR2B//PHACTR2//A2ML1//WFDC13//SPOCK1//RAPGEF4//RAB3GAP1//ARHGEF4//RALGPS2//SOS2//LAMTOR3//PSD//CSF2RA//EREG//RASGEF1A//IL2//IL5//MET//BTC//RAPGEF2//IQGAP2//CPEB2//SLIT2//ARAP2//APPL1//AGFG1//ARHGAP1//RP2//EVI5//ARHGAP12//USP6NL//EDN1//IFNA1//IFNG//INHBB//IL36G//BMP3//TNF//WNT7A//AIMP1//C1QTNF9//IGF1//ADCYAP1//CALCB//CRISP1//HPCAL4//STIM2//NRXN1//CCL28//CCL2//CCL8//MACC1//NTF3//OGN//VEGFB//PLAA//CCNY//NOS1//DENND1B//SBF2//LRP6//CALM2//UBXN2B//PPP1R15B//MTMR9//ESR1//SPRED1//EFNA5//PATE1//AGTR2// |
| GO:0000980 | RNA polymerase II distal enhancer sequence-specific DNA binding | Molecular function | 6 | 75 | 582 | 17548 | 2.41209621993127 | 0.038066931614115 | 0.60757418565272 | 1.41945212809142 | 0.0103092783505155 | ARX//PTF1A//GATA2//MEOX2//POU4F1//SMARCD2// |
| GO:0042802 | identical protein binding | Molecular function | 68 | 1661 | 582 | 17548 | 1.2343659162803 | 0.0399142003264012 | 0.60757418565272 | 1.39887256731515 | 0.116838487972509 | CALCOCO2//CEBPB//DYNLL2//SYT6//GADD45A//FMR1//CADM2//GPD1//GRIK2//GSTM3//HPRT1//INHBB//RBM44//FIBIN//LRP6//MGST1//NEUROG1//NR4A2//CHMP3//ZBTB4//BCL2A1//CLIP1//DMRTA1//BNIP3//SNX2//SOD1//VEGFB//BHLHE41//TRIM8//SBF2//GNPTG//BHLHE40//ACVR1//AIMP1//SLIT2//CD69//TCERG1//CLCN5//CRKL//APCDD1//DPYSL2//EPS15//ESR1//ARL6IP1//APPL1//HSPB8//GJB2//HSBP1//C1QTNF9//LDLR//MET//MYD88//ST8SIA3//RAB9B//POU3F2//PPARG//ATG16L1//WRNIP1//ZBTB26//PTPRG//MID1IP1//RBBP8//ATXN1//TNF//ZNF3//DCTPP1//CD84//NMI// |
| GO:0005070 | SH3/SH2 adaptor activity | Molecular function | 5 | 57 | 582 | 17548 | 2.64484234641587 | 0.0401932225207514 | 0.60757418565272 | 1.39584717253713 | 0.00859106529209622 | CRKL//ARHGAP1//NCK1//SH3BGR//SH3BGRL// |
| GO:0019904 | protein domain specific binding | Molecular function | 32 | 698 | 582 | 17548 | 1.38229009738182 | 0.0406764347790949 | 0.60757418565272 | 1.39065711926009 | 0.0549828178694158 | EPS15//CD2AP//PTPN22//OSTF1//GJA1//ARHGAP1//ENAH//SH3BGR//SH3BGRL//ADAM12//CBL//QKI//GRIK2//FZD3//RAPGEF2//PPARG//NFE2//PMEPA1//GATA2//AR//MYD88//HOXA3//CCT6A//CALM2//DDX6//ARFIP1//NCK1//ARHGEF4//HPCAL4//PRKAR2B//SKP1//BHLHE40// |
| GO:0005088 | Ras guanyl-nucleotide exchange factor activity | Molecular function | 14 | 251 | 582 | 17548 | 1.68174039238236 | 0.0408363397461591 | 0.60757418565272 | 1.38895319164106 | 0.0240549828178694 | ARHGEF4//SOS2//RAPGEF2//DENND1B//RAB3GAP1//SBF2//RAPGEF4//CSF2RA//EREG//RASGEF1A//IL2//IL5//MET//BTC// |
| GO:0004955 | prostaglandin receptor activity | Molecular function | 2 | 10 | 582 | 17548 | 6.03024054982818 | 0.0414215692445018 | 0.60757418565272 | 1.38277345200659 | 0.00343642611683849 | PTGFR//PPARG// |
| GO:0015279 | store-operated calcium channel activity | Molecular function | 2 | 10 | 582 | 17548 | 6.03024054982818 | 0.0414215692445018 | 0.60757418565272 | 1.38277345200659 | 0.00343642611683849 | STIM2//TRPC3// |
| GO:0015467 | G-protein activated inward rectifier potassium channel activity | Molecular function | 2 | 10 | 582 | 17548 | 6.03024054982818 | 0.0414215692445018 | 0.60757418565272 | 1.38277345200659 | 0.00343642611683849 | KCNJ3//KCNJ10// |
| GO:0016723 | oxidoreductase activity, oxidizing metal ions, NAD or NADP as acceptor | Molecular function | 2 | 10 | 582 | 17548 | 6.03024054982818 | 0.0414215692445018 | 0.60757418565272 | 1.38277345200659 | 0.00343642611683849 | STEAP2//MTRR// |
| GO:0030957 | Tat protein binding | Molecular function | 2 | 10 | 582 | 17548 | 6.03024054982818 | 0.0414215692445018 | 0.60757418565272 | 1.38277345200659 | 0.00343642611683849 | DLL1//MDFIC// |
| GO:0005085 | guanyl-nucleotide exchange factor activity | Molecular function | 17 | 323 | 582 | 17548 | 1.58690540784952 | 0.0417238445394229 | 0.60757418565272 | 1.37961568142166 | 0.0292096219931271 | PSD//RAPGEF4//CSF2RA//EREG//RASGEF1A//IL2//IL5//MET//BTC//RAPGEF2//ARHGEF4//SOS2//DENND1B//RAB3GAP1//SBF2//RALGPS2//LAMTOR3// |
| GO:0030159 | receptor signaling complex scaffold activity | Molecular function | 3 | 24 | 582 | 17548 | 3.76890034364261 | 0.0438038166161517 | 0.623843366313545 | 1.35848804787212 | 0.00515463917525773 | NCK1//DLG5//G3BP2// |
| GO:0030552 | cAMP binding | Molecular function | 3 | 24 | 582 | 17548 | 3.76890034364261 | 0.0438038166161517 | 0.623843366313545 | 1.35848804787212 | 0.00515463917525773 | RAPGEF4//PRKAR2B//RAPGEF2// |
| GO:0004527 | exonuclease activity | Molecular function | 6 | 78 | 582 | 17548 | 2.31932328839545 | 0.0447578089969335 | 0.6284061786408 | 1.34913118138925 | 0.0103092783505155 | CNOT7//CNOT6//DCP2//CNOT6L//PAN3//RAD9B// |
| GO:0019003 | GDP binding | Molecular function | 5 | 59 | 582 | 17548 | 2.55518667365601 | 0.0455788432038852 | 0.6284061786408 | 1.34123670145807 | 0.00859106529209622 | RAB12//RAB9B//RAP2C//RRAGD//RAB28// |
| GO:0097110 | scaffold protein binding | Molecular function | 5 | 59 | 582 | 17548 | 2.55518667365601 | 0.0455788432038852 | 0.6284061786408 | 1.34123670145807 | 0.00859106529209622 | DYNLL2//GJA1//DLL1//NOS1//TREM1// |
| GO:0019210 | kinase inhibitor activity | Molecular function | 7 | 99 | 582 | 17548 | 2.13190322468673 | 0.046413481833957 | 0.633177604808508 | 1.33335585057355 | 0.0120274914089347 | FLRT1//NCK1//SOCS5//CASP3//PRKAR2B//SPRED1//LRP6// |
| GO:0019201 | nucleotide kinase activity | Molecular function | 3 | 25 | 582 | 17548 | 3.61814432989691 | 0.0485901863979854 | 0.635628369065309 | 1.31345143492627 | 0.00515463917525773 | AK4//CMPK1//DLG2// |
| GO:1901567 | fatty acid derivative binding | Molecular function | 3 | 25 | 582 | 17548 | 3.61814432989691 | 0.0485901863979854 | 0.635628369065309 | 1.31345143492627 | 0.00515463917525773 | ACBD3//ACBD5//PPARG// |
| GO:0004143 | diacylglycerol kinase activity | Molecular function | 2 | 11 | 582 | 17548 | 5.48203686348016 | 0.0495358528361082 | 0.635628369065309 | 1.30508035555773 | 0.00343642611683849 | DGKH//DGKE// |
| GO:0004954 | prostanoid receptor activity | Molecular function | 2 | 11 | 582 | 17548 | 5.48203686348016 | 0.0495358528361082 | 0.635628369065309 | 1.30508035555773 | 0.00343642611683849 | PPARG//PTGFR// |
| GO:0005172 | vascular endothelial growth factor receptor binding | Molecular function | 2 | 11 | 582 | 17548 | 5.48203686348016 | 0.0495358528361082 | 0.635628369065309 | 1.30508035555773 | 0.00343642611683849 | VEGFB//CD2AP// |
| GO:0043295 | glutathione binding | Molecular function | 2 | 11 | 582 | 17548 | 5.48203686348016 | 0.0495358528361082 | 0.635628369065309 | 1.30508035555773 | 0.00343642611683849 | GSTM3//MGST1// |
